# Standardisation of data from real-time quantitative PCR methods – evaluation of outliers and comparison of calibration curves

**DOI:** 10.1186/1472-6750-5-31

**Published:** 2005-12-07

**Authors:** Malcolm J Burns, Gavin J Nixon, Carole A Foy, Neil Harris

**Affiliations:** 1 Bio-Molecular Innovation, LGC Limited, Queens Road, Teddington, Middlesex, TW11 0LY, UK

## Abstract

**Background:**

As real-time quantitative PCR (RT-QPCR) is increasingly being relied upon for the enforcement of legislation and regulations dependent upon the trace detection of DNA, focus has increased on the quality issues related to the technique. Recent work has focused on the identification of factors that contribute towards significant measurement uncertainty in the real-time quantitative PCR technique, through investigation of the experimental design and operating procedure. However, measurement uncertainty contributions made during the data analysis procedure have not been studied in detail. This paper presents two additional approaches for standardising data analysis through the novel application of statistical methods to RT-QPCR, in order to minimise potential uncertainty in results.

**Results:**

Experimental data was generated in order to develop the two aspects of data handling and analysis that can contribute towards measurement uncertainty in results. This paper describes preliminary aspects in standardising data through the application of statistical techniques to the area of RT-QPCR. The first aspect concerns the statistical identification and subsequent handling of outlying values arising from RT-QPCR, and discusses the implementation of ISO guidelines in relation to acceptance or rejection of outlying values. The second aspect relates to the development of an objective statistical test for the comparison of calibration curves.

**Conclusion:**

The preliminary statistical tests for outlying values and comparisons between calibration curves can be applied using basic functions found in standard spreadsheet software. These two aspects emphasise that the comparability of results arising from RT-QPCR needs further refinement and development at the data-handling phase. The implementation of standardised approaches to data analysis should further help minimise variation due to subjective judgements. The aspects described in this paper will help contribute towards the development of a set of best practice guidelines regarding standardising handling and interpretation of data arising from RT-QPCR experiments.

## Background

Real-time quantitative PCR (RT-QPCR) is increasingly being seen as a bench-marking analytical tool for many trace DNA detection strategies, across a diverse range of areas encompassed within bioanalytical science [1-5] High quality performance characteristics associated with this technique include throughput, reproducibility, specificity, and sensitivity. These characteristics in association with its wide applicability [6,7], have meant that RT-QPCR is now being seen as a 'gold standard' for comparative purposes across a number of disciplines inclusive of regulation and legislation [8-10]

Because of the wide applicability of RT-QPCR, there is now a wealth of information pertaining to the analytical results derived from this molecular technique [11,12] For this reason, it is imperative that the performance characteristics and uncertainty contributions from a particular application of RT-QPCR are known with high confidence. Without these defined criteria, the method cannot be qualified as performing 'fit for purpose', and speculation may arise regarding the interpretation and assurance with which results are derived [13].

For a given assay, measurement uncertainty estimation helps identify components of variability and make reasonable estimates of these components' effects upon the end result [2,13] Current quality regulations dictate that any result from an analytical laboratory should be given with an associated uncertainty estimate, and this is now included under the remit of ISO 17025 [14-16]. In relation to RT-QPCR, much of the current focus of measurement uncertainty estimation is based upon identifying factors associated with the experimental conditions of the analytical technique [17-20]. For example, uncertainty in RT-QPCR can arise from the use of laboratory equipment and reagent preparation associated with the initial DNA extraction procedure.

Additionally, recent studies have examined the underlying mathematical model associated with RT-QPCR, inclusive of the precision of replicate standard curves [21]. Progressing from this, the use of a sigmoidal function to model fluorescence data as a more reliable alternative to using standard curves has been proposed [22]. Aspects associated with the in-house validation of RT-QPCR measurements have also been examined [23,24], and some of the pertinent factors that account for a lot of the variation in RT-QPCR measurements have been reviewed [25].

A critical aspect that has not been examined in great detail is the area of data handling and interpretation. The vast volume of data being generated by RT-QPCR means that a large number of statistical tools and approaches can be applied to analyse the results [26]. However, if there are no guidelines or standardised approaches for this data handling and interpretation, then significant variation in the end result can also be attributed to this area. As one of the functions of measurement uncertainty estimation is to identify all potential factors that contribute towards the variation in the end result, then the area of data handling and interpretation is a fundamental aspect that should also be examined in detail.

Previous studies have identified production of calibration curves, interpretation of data from duplex and singleplex reactions, and transformation of data, as areas that can contribute towards variation in the interpretation of RT-QPCR data [2]. The current study contributes further towards standardised methodologies that can be implemented when results are interpreted from trace detection situations. In the current paper, two additional aspects of data analysis are presented, which can potentially give rise to different interpretations of results from real-time PCR experiments if standardised guidelines are not adhered to in their implementation.

The first aspect concerns the identification and subsequent handling of outlying values. Inclusion of outlying values in a data set is liable to give rise to erroneous interpretations. For many data sets arising from analytical procedures, it is often advantageous to display the distribution of the data set visually, to aid in the identification of potential outliers. There are also a number of statistical tools available that facilitate an objective test as to whether a data point should be classified as outlying [27].

However, there are inherent difficulties associated with conducting analysis of data arising from RT-QPCR [2]. Some of these problems originate from the artificially imposed end cycle number, which represents the total number of amplification cycles performed on the RT-QPCR platforms. The rather arbitrary assignment of this value coupled with the non-normal distribution of blank controls and data points that lie close to this value can make the identification of outlying values problematic. The establishment of guidelines on how to identify and handle outlying values given the regulatory and legislative dimension that RT-QPCR now occupies, is thus of fundamental importance in minimising potential measurement uncertainty.

This paper describes a simple visual approach as an initial step to identifying potential outlying values using a 'box and whisker' plot, and then suggests the use of a statistical test to objectively assess values to determine whether the data points are outliers. Additionally, the implementation of ISO guidelines is discussed in relation to acceptance or rejection of statistical outliers.

The second aspect relating to analysis of data arising from RT-QPCR concerns the comparison of calibration curves. Calibration curves are produced based on measuring an instrument response according to a range of standards of known analyte concentration. For RT-QPCR studies, the DNA content of an unknown sample is then estimated by translating the value of the measurand associated with a sample into its corresponding DNA concentration based upon the equation relating to the calibration curve.

Calibration curves arising from PCR are often compared to one another in order to judge if they are performing the same. One way of doing this is to examine the magnitude of the regression coefficient associated with the calibration curve produced through simple linear regression. This regression coefficient is equal to the gradient or slope of the line and is related to the efficiency of PCR amplification. The current state of the art in RT-QPCR for comparison of calibration curves is to do so visually in order to assess any difference between these regression coefficients [28]. This visual comparison is potentially very subjective, and there is a need for an objective statistical test that will indicate the likelihood of two regression coefficients being equal. Such a test is described in detail in this paper so that it can be applied using basic functions found in standard spreadsheet software, and the implementation of its principles should further help minimise variation due to probable subjective judgements.

This paper further highlights original applications of statistical tools to the analysis of data arising from RT-QPCR techniques. Approaches for identifying and handling outlying values, and techniques for comparing calibration curves, are examined. These aspects are discussed with a view to helping minimise potential measurement uncertainty arising from the area of RT-QPCR data analysis. The analysis of these aspects illustrates preliminary methods in which to standardise the reporting of results. To fully explore and model the optimal approach to data handling from RT-QPCR requires a collaborative effort between scientists and statisticians alike. It is hoped that the approaches outlined here will contribute towards the development of a set of best practice guidelines with which to help standardise handling and interpretation of data arising from RT-QPCR.

## Results and discussion

### Outlier testing

A preliminary method to identify potential outliers is presented here, based on initially displaying data sets as graphical box and whisker plots [29]. These graphs were produced using Statistica 6.0 software (Statsoft Inc., USA) but can be reproduced using other statistical software packages.

The rationale behind the box and whisker plot was as follows. The midpoint of a data set was calculated and represented by the median. A box drawn around this midpoint represented the inter-quartile range, which encompassed 50% of the range based on the 1^st ^quartile (25% confidence level) to the 3^rd ^quartile (75% confidence level). The whiskers outside of the box represented an additional selected range, encompassing 5% to 95% of the range of results. Data points that lay substantially beyond the range of this box and whisker plot were identified as potential outliers. Typically, potential outliers were identified when the value associated with a data point was larger than an upper limit of 1.5 times the height of the box, or below the lower limit of 1.5 time the height of the box.

A more formal definition is outlined below:

Upper limit = (75^th ^percentile) + (outlier coefficient*(75^th ^percentile – 25^th ^percentile))

Lower limit = (25^th ^percentile) - (outlier coefficient*(75^th ^percentile – 25^th ^percentile))

The default setting for the outlier coefficient is 1.5.

If the value associated with a data point was above the upper limit, or below the lower limit, it is thus characterised as a potential outlier.

**Figure 1:**
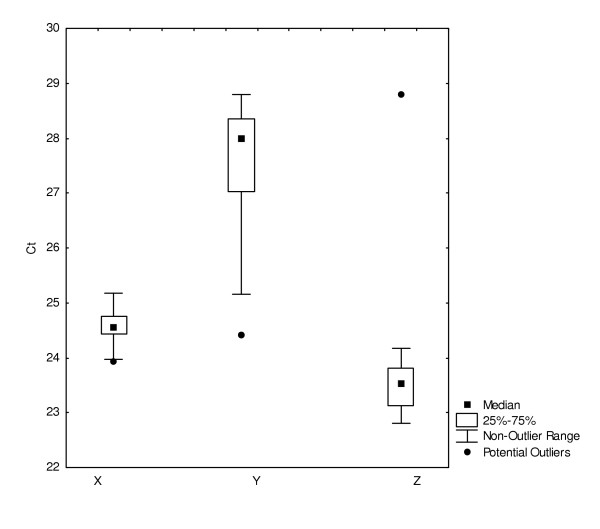
**Box and whisker plots applied to data set 3 to identify potential outliers**. The three samples of X, Y and Z are represented on the graph. The response variable on the y-axis is represented by Ct (cycle threshold value). For each sample, the median is indicated as the smaller box, and the large box indicates the inter-quartile range (1^st ^to 3^rd ^quartiles). Box and whiskers encompass 95% of the range of the data associated with each sample.

Data set 3 was produced based on measuring cycle threshold (Ct) values associated with three samples X, Y, and Z, which consisted of 18 replicate observations per sample. The Ct value indicated the cycle number where the target analyte signal crossed a pre-set threshold value during RT-QPCR. The graph in Figure 1 shows the results from data set 3 that has been displayed according to a box and whisker plot, using the description outlined above where the whiskers encompass 5% to 95% of the range of results.

An outlier can be defined as a data point that does not follow the typical distribution of the rest of the data set and can be regarded as an irregular observation. Example causes of outlying data points include the sample being atypical, the underlying distribution of the data set being non-normal in nature, operator error, a measurement mistake or transcription error, or purely due to chance variation. Potential outliers can arise due to this latter chance variation, where the data point is correct in nature and is simply more divergent than the majority of the data set, or they can arise due to errors where the value of the data point is erroneous. An objective test is needed to calculate the probability that a single data point is different from the rest of the data set purely due to chance alone.

The use of the "box and whisker" plot is a useful diagnostic aid in achieving this, and a number of further statistical tools exist which can be used in conjunction to conduct this objective test [30]. The Grubbs' test [31] for identification of outlying values will be outlined here due to its ease of application, computational simplicity, and its recommendation as described in the International Standard Organisation document ISO 5725-2 [32].

Alternative names for the Grubbs' test are the maximum normalised residual test and the extreme studentized deviate.

The null hypothesis for the Grubbs' test is that there are no outliers in the data set, whilst the alternative hypothesis is that there is at least one outlier present. The test statistic for the Grubbs' test is computed from:

G = max [Yi−Y¯]sd
 MathType@MTEF@5@5@+=feaafiart1ev1aaatCvAUfKttLearuWrP9MDH5MBPbIqV92AaeXatLxBI9gBaebbnrfifHhDYfgasaacH8akY=wiFfYdH8Gipec8Eeeu0xXdbba9frFj0=OqFfea0dXdd9vqai=hGuQ8kuc9pgc9s8qqaq=dirpe0xb9q8qiLsFr0=vr0=vr0dc8meaabaqaciGacaGaaeqabaqabeGadaaakeaacqqGhbWrcqqGGaaicqqG9aqpcqqGGaaicqqGTbqBcqqGHbqycqqG4baEcqqGGaaidaWcaaqaaiabbUfaBjabdMfaznaaBaaaleaacqWGPbqAaeqaaOGaeyOeI0IafmywaKLbaebacqGGDbqxaeaacqqGZbWCcqqGKbazaaaaaa@3F91@

Where:

G = test statistic associated with Grubb's test

Y*i *= *ith *observation from data set / suspected outlier

Y¯
 MathType@MTEF@5@5@+=feaafiart1ev1aaatCvAUfKttLearuWrP9MDH5MBPbIqV92AaeXatLxBI9gBaebbnrfifHhDYfgasaacH8akY=wiFfYdH8Gipec8Eeeu0xXdbba9frFj0=OqFfea0dXdd9vqai=hGuQ8kuc9pgc9s8qqaq=dirpe0xb9q8qiLsFr0=vr0=vr0dc8meaabaqaciGacaGaaeqabaqabeGadaaakeaacuWGzbqwgaqeaaaa@2E01@ = sample mean

sd = standard deviation of data set

The test statistic can be interpreted as the largest absolute deviation from the sample mean in terms of the sample standard deviation.

It is possible to calculate the probability associated with the test statistic using formulae, but it is more common to determine the critical values associated with the test statistic using tables available in statistical publications [31]. We have used tables to determine the probability associated with the test statistic, so that the approach for identifying outliers can be applied using standard spreadsheet software rather than specialist statistical software.

Based on ISO 5725 guidelines, outlying data points can be characterised according to the probability that their associated values can arise due to chance alone. Those values, which lie between 95% and 99% of the expected range of the characterised distribution, are termed stragglers (P value between 5% and 1%), and those values, which lie beyond 99% of the range of the characterised distribution, are termed outliers (P value below 1%).

From the box and whiskers plot illustrated in Figure 1, potential outliers were observed in all three samples of X, Y and Z. The most extreme potential outlier was observed in sample Z and the least extreme potential outlier in sample X. The Grubbs' test was applied to each of the samples as an objective assessment as to whether these data points should be classified as outliers or stragglers according to ISO guidlines. The results of the Grubbs' test are shown in Table 1.

For sample X, the test statistic associated with the potential outlier was 1.89 based on 18 data points. According to statistical tables [31] in order for the extreme value to be a straggler (beyond 95% of the range) the test statistics must be 2.50 or higher for 18 observations. This implied that the extreme value was not inherently different from the majority of values for sample X, and was within the 95% confidence interval of the mean of that data set. Thus the extreme value was included for subsequent analysis, as it was not considered inherently different from the rest of the data set.

For sample Y, the potential outlying value has a response value (Ct) of 24.41, which appeared distinct from the rest of the range of the distribution for sample Y. The Grubbs' test statistic for this value was 2.63, which indicated that it lay between 95% and 99% of the normal range of the sample Y based on statistical tables. According to ISO guidelines this should be considered as a straggler.

The extreme value in sample Z had an associated Ct value of 28.79. The Grubbs' test statistic associated with this data point was 3.83 that indicated that the value lay beyond 99% of the range of sample Z, given a normal distribution. This value was thus considered as an outlier according to ISO guidelines.

ISO 5725 provides recommendations regarding the handling of potential outliers [32]. It suggests retention of stragglers in a data set unless there is a technical reason not to do so, based on the rationale that at the 95% level of confidence, there is a reasonable probability (5%) that the straggler could arise from the data set purely due to chance alone. However, for data points classified as outliers, ISO guidelines recommend rejection of the value before the subsequent data analysis, unless sufficient justification is given to retain it. This is based on the premise that there is an unacceptably high chance that the value does not belong to the rest of the data set.

Retention of statistical outliers in a data set can cause the mean value to be changed slightly whilst the confidence interval can be greatly increased. For example, when the single outlier has been removed from sample Z (Table 1), the mean and standard deviation associated with the data set are 23.46 and 0.40 respectively. However, inclusion of the single outlier gives a mean of 23.75 and a standard deviation of 1.32. This effect is particularly important not only if the sample is being used to construct a calibration curve, but also if that sample is being analysed in order to estimate an unknown analyte level associated with it.

There are potential difficulties associated with conducting the analysis of data arising from RT-QPCR. Many of these difficulties arise from the artificially imposed end cycle number. This end cycle number represents the maximum number of cycles that the RT-QPCR platform is instructed to perform for a given assay. Results arising from RT-QPCR platforms are typically expressed as cycle threshold values (Ct values). Because of the nature of RT-QPCR, blank control samples consisting of no template controls do not give a zero Ct value, but instead give a response value that is equal to the end cycle number. Samples that fail to amplify, or ones that are true blanks, will have a mean equal to the end cycle number with a standard deviation of zero. Potentially this can cause problems when visually inspecting a data set for outlying values, as data points near the end cycle number may be wrongly rejected when they represent true values. This is of particular importance when estimating limits of detection and quantification associated with an assay where the confidence associated with a reported result may be in question.

Aside from the statistical test outlined here, there are a number of approaches that can be implemented in order to accommodate or reject any data points that could be considered outliers. For example, one approach is to use the unadulterated data in order to assess the performance of different PCR platforms. This is based on the premise that if the same quality regulations are adhered to in the operation of each platform, the number of erroneous data points in each data set will be indicative of the overall performance of that platform. A further approach is to artificially impose a maximum Ct value, above which any values will be rejected based on the risk of being outlying values, resulting from non-specific amplification, for example. Dependent upon the capabilities of the real-time PCR platform, this approach has the potential to incorrectly omit values that validly add information to the analysis. An additional approach includes the use of experience to identify potential outliers, which can be extremely subjective and variable in nature, but is arguably a method of practical use. Other approaches exist to improve the quality of data arising from RT-QPCR, many of these being subjective in nature or exhibiting a potentially high chance of incorrectly rejecting data points that would add value to the analysis. The statistical procedure and approach outlined above provides a basis for more objective determination of when to accept or reject potential outlying data points.

### Limitations of approach and comparison to other methods

A limitation of the Grubbs' test is that the test assumes that the data set follows an approximately normal distribution. The user should first test their data set for significant departure from normality before proceeding. Tests such as the Shapiro-Wilk W test [33] and Lilliefors test [34] can be used to test for normality. Transformation of the raw data values into a mathematical derivative, for example through using logarithms, may help normalise the data set and make it amenable for further parametric statistics [35]. Outlier tests for non-normally distributed data can be applied, but the power of these tests is relatively poor, and they are more difficult to apply.

The particular form of the Grubbs' test illustrated in this paper detects one statistical outlier at a time. Alternative tests for single outliers exist, for example Dixon's Q test [36], although the Grubbs' test is usually considered to be more robust, and the Grubbs' test is recommended as an applicable outlier test according to the International Standard Organisation guidelines ISO 5725-2 [32].

The Grubbs test is only valid for the detection of two or less outliers in a data set. Derivations of the Grubb's test also exist for detecting pairs of outlying values, but a discussion of these is beyond the remit of the current article. It is possible to use the Grubbs' test iteratively on the remainder of the data set in order to identify all potential outliers. However, such multiplicity of testing decreases the power of the Grubbs' test and this use of repeated significance tests in a single study can thus increase the probability of obtaining false positive results. Statistical techniques for handling the iterative elimination of outliers can be used, and for identification of multiple outliers within a data set that consists of 25 or more values that follow a normal distribution, it is recommended that further outlier tests such as the Rosner's test be considered [30].

### Comparing regression coefficients

For RT-QPCR, the majority of calibration curves are fitted using a simple linear regression model, although alternative models are available [2,37,38]. This simple linear regression model uses the method of least squares, which establishes the best fitting straight line based on minimising the residual variance between the predicted model and the observed data points. The resulting linear regression equation is often displayed in the form of y = bx + c, where y is the dependent variable, x is the independent variable, b is the gradient of the line, and c is the intercept of the line on the y-axis. Comparisons between different calibration curves are usually conducted based upon using the regression coefficient. This regression coefficient (b) represents the gradient, or slope of the line, associated with the calibration curve, and is also related to the amplification efficiency of the real-time PCR reaction. For many RT-QPCR applications, an assumption is made that the PCR efficiency of the standards used to construct the calibration curve is the same as the PCR efficiency associated with the samples under evaluation. Thus, comparison of regression coefficients is an important quality step. This regression coefficient corresponds to the gradient of the rate of change of the dependent variable (y) per unit change in the independent variable (x). The most common way of comparing calibration curves together for real-time PCR is to inspect the gradient of the two regression lines visually on the same or separate graphs [28]. However, this only provides a subjective assessment of the differences between the two lines.

Outlined below is an objective statistical test that calculates the probability that any differences observed between two regression coefficients are due to chance alone.

This test is a simplified derivative of an analysis of covariance. The analysis of covariance is recommended as the best choice for comparing regression lines [39], and a review and explanation of the full procedure are given in [40,41]. The test is explained in detail using data sets 1 and 2 so that the computations involved in the worked example can be readily implemented using only basic spreadsheet software commonly available to the bio-analytical community.

The validity of this test is based upon the following assumptions:

• Simple linear regression is used to produce the calibration curves

• Each of the two data sets have the same values for the independent variable x

• Variances between the data sets are statistically equal

**Figure 2:**
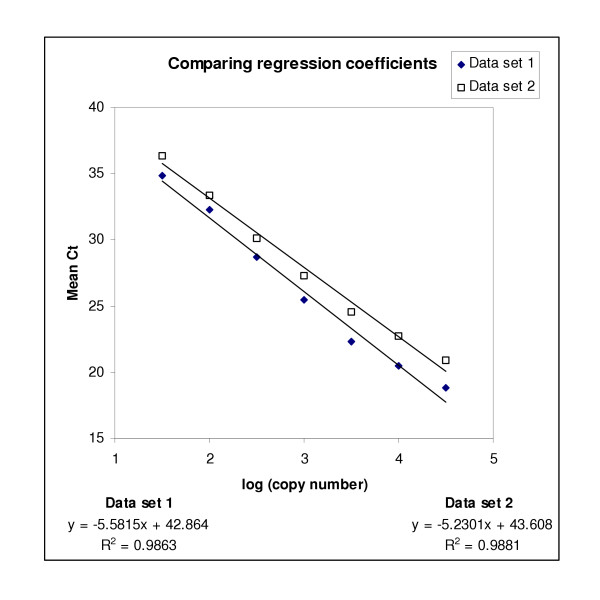
**Calibration curves associated with data sets 1 and 2**. Calibration curves are produced by simple linear regression based on values from seven standards. Regression equations, R^2 ^values and regression coefficients associated with both data sets are shown towards the bottom of the graph.

Data sets 1 and 2 are described in Table 2. These were analysed by simple linear regression as shown in Table 3 in order to produce two calibration curves (Figure 2). This resulted in two estimates of the regression coefficients (b_1 _and b_2_) associated with each of the calibration curves for data set 1 and 2 respectively. From this analysis:

b_1 _= -5.5815

b_2 _= -5.2301

These two regression coefficient estimates are similar, but the magnitude of the regression coefficient b_1 _is slightly larger than b_2_. Figure 2 also illustrates that the two regression lines appear to converge at low copy number values, whilst they diverge at higher copy number values.

The objective test for differences between the two regression coefficients uses the calculation of a term called the heterogeneity of regression coefficients. This heterogeneity of regression coefficients tests the null hypothesis that b_1 _and b_2 _are estimates of the same gradient. This can be calculated using the following formulae:

Heterogeneity of regression coefficients SS = ∑i=1n
 MathType@MTEF@5@5@+=feaafiart1ev1aaatCvAUfKttLearuWrP9MDH5MBPbIqV92AaeXatLxBI9gBaebbnrfifHhDYfgasaacH8akY=wiFfYdH8Gipec8Eeeu0xXdbba9frFj0=OqFfea0dXdd9vqai=hGuQ8kuc9pgc9s8qqaq=dirpe0xb9q8qiLsFr0=vr0=vr0dc8meaabaqaciGacaGaaeqabaqabeGadaaakeaadaaeWbqaaaWcbaGaemyAaKMaeyypa0JaeGymaedabaGaemOBa4ganiabggHiLdaaaa@33A6@ (Regression SS) – (Joint Regression SS) where 'SS' is equal to the Sum of Squares.

The ∑i=1n
 MathType@MTEF@5@5@+=feaafiart1ev1aaatCvAUfKttLearuWrP9MDH5MBPbIqV92AaeXatLxBI9gBaebbnrfifHhDYfgasaacH8akY=wiFfYdH8Gipec8Eeeu0xXdbba9frFj0=OqFfea0dXdd9vqai=hGuQ8kuc9pgc9s8qqaq=dirpe0xb9q8qiLsFr0=vr0=vr0dc8meaabaqaciGacaGaaeqabaqabeGadaaakeaadaaeWbqaaaWcbaGaemyAaKMaeyypa0JaeGymaedabaGaemOBa4ganiabggHiLdaaaa@33A6@ (Regression SS) is equal to the sum of squares associated with the regression item, where the Sigma symbol is used to indicate that this is summed across both data sets 1 and 2. These values relate to the regression items from the analysis of variance tables which are produced in the original regression analysis conducted on data sets 1 and 2, as illustrated in Table 3. The Regression SS characterises the component of the variation in the dependent variable (y) that is accountable by the independent variable (x).

From Table 3, the Regression SS for data set 1 was 217.6061, and the Regression SS for data set 2 was 190.9423. Therefore the ∑i=1n
 MathType@MTEF@5@5@+=feaafiart1ev1aaatCvAUfKttLearuWrP9MDH5MBPbIqV92AaeXatLxBI9gBaebbnrfifHhDYfgasaacH8akY=wiFfYdH8Gipec8Eeeu0xXdbba9frFj0=OqFfea0dXdd9vqai=hGuQ8kuc9pgc9s8qqaq=dirpe0xb9q8qiLsFr0=vr0=vr0dc8meaabaqaciGacaGaaeqabaqabeGadaaakeaadaaeWbqaaaWcbaGaemyAaKMaeyypa0JaeGymaedabaGaemOBa4ganiabggHiLdaaaa@33A6@ (Regression SS) was equal to 408.5484.

The Joint Regression SS uses the Sum of the Products (SP) and is calculated as:

Joint Regression SS = (∑i=1nSP[x,y])2∑i=1nSS[x]
 MathType@MTEF@5@5@+=feaafiart1ev1aaatCvAUfKttLearuWrP9MDH5MBPbIqV92AaeXatLxBI9gBaebbnrfifHhDYfgasaacH8akY=wiFfYdH8Gipec8Eeeu0xXdbba9frFj0=OqFfea0dXdd9vqai=hGuQ8kuc9pgc9s8qqaq=dirpe0xb9q8qiLsFr0=vr0=vr0dc8meaabaqaciGacaGaaeqabaqabeGadaaakeaadaWcaaqaamaabmaabaWaaabCaeaacqWGtbWucqWGqbaudaWgaaWcbaGaei4waSLaemiEaGNaeiilaWIaemyEaKNaeiyxa0fabeaaaeaacqWGPbqAcqGH9aqpcqaIXaqmaeaacqWGUbGBa0GaeyyeIuoaaOGaayjkaiaawMcaamaaCaaaleqabaGaeGOmaidaaaGcbaWaaabCaeaacqWGtbWucqWGtbWudaWgaaWcbaGaei4waSLaemiEaGNaeiyxa0fabeaaaeaacqWGPbqAcqGH9aqpcqaIXaqmaeaacqWGUbGBa0GaeyyeIuoaaaaaaa@4CAF@

Where ∑i=1n
 MathType@MTEF@5@5@+=feaafiart1ev1aaatCvAUfKttLearuWrP9MDH5MBPbIqV92AaeXatLxBI9gBaebbnrfifHhDYfgasaacH8akY=wiFfYdH8Gipec8Eeeu0xXdbba9frFj0=OqFfea0dXdd9vqai=hGuQ8kuc9pgc9s8qqaq=dirpe0xb9q8qiLsFr0=vr0=vr0dc8meaabaqaciGacaGaaeqabaqabeGadaaakeaadaaeWbqaaaWcbaGaemyAaKMaeyypa0JaeGymaedabaGaemOBa4ganiabggHiLdaaaa@33A6@ SP_[x, y] _is the Sum of the Products for x and y summed across both data sets, and ∑i=1n
 MathType@MTEF@5@5@+=feaafiart1ev1aaatCvAUfKttLearuWrP9MDH5MBPbIqV92AaeXatLxBI9gBaebbnrfifHhDYfgasaacH8akY=wiFfYdH8Gipec8Eeeu0xXdbba9frFj0=OqFfea0dXdd9vqai=hGuQ8kuc9pgc9s8qqaq=dirpe0xb9q8qiLsFr0=vr0=vr0dc8meaabaqaciGacaGaaeqabaqabeGadaaakeaadaaeWbqaaaWcbaGaemyAaKMaeyypa0JaeGymaedabaGaemOBa4ganiabggHiLdaaaa@33A6@ SS_[x] _is the Sum of Squares of x summed across both data sets. Within a data set, these terms are further defined as follows:

SP[x,y]=∑i=1n(xi−x¯)(yi−y¯)
 MathType@MTEF@5@5@+=feaafiart1ev1aaatCvAUfKttLearuWrP9MDH5MBPbIqV92AaeXatLxBI9gBaebbnrfifHhDYfgasaacH8akY=wiFfYdH8Gipec8Eeeu0xXdbba9frFj0=OqFfea0dXdd9vqai=hGuQ8kuc9pgc9s8qqaq=dirpe0xb9q8qiLsFr0=vr0=vr0dc8meaabaqaciGacaGaaeqabaqabeGadaaakeaacqWGtbWucqWGqbaudaWgaaWcbaWaamWaaeaacqWG4baEcqGGSaalcqWG5bqEaiaawUfacaGLDbaaaeqaaOGaeyypa0ZaaabCaeaadaqadaqaaiabdIha4naaBaaaleaacqWGPbqAaeqaaOGaeyOeI0IafmiEaGNbaebaaiaawIcacaGLPaaaaSqaaiabdMgaPjabg2da9iabigdaXaqaaiabd6gaUbqdcqGHris5aOWaaeWaaeaacqWG5bqEdaWgaaWcbaGaemyAaKgabeaakiabgkHiTiqbdMha5zaaraaacaGLOaGaayzkaaaaaa@4B30@

and

SS[x]=∑i=1n(xi−x¯)2
 MathType@MTEF@5@5@+=feaafiart1ev1aaatCvAUfKttLearuWrP9MDH5MBPbIqV92AaeXatLxBI9gBaebbnrfifHhDYfgasaacH8akY=wiFfYdH8Gipec8Eeeu0xXdbba9frFj0=OqFfea0dXdd9vqai=hGuQ8kuc9pgc9s8qqaq=dirpe0xb9q8qiLsFr0=vr0=vr0dc8meaabaqaciGacaGaaeqabaqabeGadaaakeaacqWGtbWucqWGtbWudaWgaaWcbaWaamWaaeaacqWG4baEaiaawUfacaGLDbaaaeqaaOGaeyypa0ZaaabCaeaadaqadaqaaiabdIha4naaBaaaleaacqWGPbqAaeqaaOGaeyOeI0IafmiEaGNbaebaaiaawIcacaGLPaaaaSqaaiabdMgaPjabg2da9iabigdaXaqaaiabd6gaUbqdcqGHris5aOWaaWbaaSqabeaacWaGacaaKdaIYaGmaaaaaa@44D1@

Using this formulae the SP_[x, y] _for data set 1 is calculated as -38.9871, and the SP_[x, y] _for data set 2 is calculated as -36.5205. Hence:

∑i=1n(SP[x,y])2=5701.389
 MathType@MTEF@5@5@+=feaafiart1ev1aaatCvAUfKttLearuWrP9MDH5MBPbIqV92AaeXatLxBI9gBaebbnrfifHhDYfgasaacH8akY=wiFfYdH8Gipec8Eeeu0xXdbba9frFj0=OqFfea0dXdd9vqai=hGuQ8kuc9pgc9s8qqaq=dirpe0xb9q8qiLsFr0=vr0=vr0dc8meaabaqaciGacaGaaeqabaqabeGadaaakeaadaaeWbqaamaabmaabaGaem4uamLaemiuaa1aaSbaaSqaamaadmaabaGaemiEaGNaeiilaWIaemyEaKhacaGLBbGaayzxaaaabeaaaOGaayjkaiaawMcaaaWcbaGaemyAaKMaeyypa0JaeGymaedabaGaemOBa4ganiabggHiLdGcdaahaaWcbeqaaiabikdaYaaakiabg2da9iabiwda1iabiEda3iabicdaWiabigdaXiabc6caUiabiodaZiabiIda4iabiMda5aaa@4764@

The denominator for the Joint regression Sum of Squares is the summation of the sum of squares for data sets 1 and 2. As both data sets use the same values associated with the independent variable x, then the Sum of Squares of x for both data sets is the same. Hence:

∑i=1nSS[x]=2∑(xi−x¯)2
 MathType@MTEF@5@5@+=feaafiart1ev1aaatCvAUfKttLearuWrP9MDH5MBPbIqV92AaeXatLxBI9gBaebbnrfifHhDYfgasaacH8akY=wiFfYdH8Gipec8Eeeu0xXdbba9frFj0=OqFfea0dXdd9vqai=hGuQ8kuc9pgc9s8qqaq=dirpe0xb9q8qiLsFr0=vr0=vr0dc8meaabaqaciGacaGaaeqabaqabeGadaaakeaadaaeWbqaaiabdofatjabdofatnaaBaaaleaadaWadaqaaiabdIha4bGaay5waiaaw2faaaqabaaabaGaemyAaKMaeyypa0JaeGymaedabaGaemOBa4ganiabggHiLdGccqGH9aqpcqaIYaGmdaaeabqaamaabmaabaGaemiEaG3aaSbaaSqaaiabdMgaPbqabaGccqGHsislcuWG4baEgaqeaaGaayjkaiaawMcaaaWcbeqab0GaeyyeIuoakmaaCaaaleqabaGaeGOmaidaaaaa@45E0@

The Sum of Squares of x for data set 1 is calculated as 6.985063, thus:

∑i=1nSS[x]=13.9701
 MathType@MTEF@5@5@+=feaafiart1ev1aaatCvAUfKttLearuWrP9MDH5MBPbIqV92AaeXatLxBI9gBaebbnrfifHhDYfgasaacH8akY=wiFfYdH8Gipec8Eeeu0xXdbba9frFj0=OqFfea0dXdd9vqai=hGuQ8kuc9pgc9s8qqaq=dirpe0xb9q8qiLsFr0=vr0=vr0dc8meaabaqaciGacaGaaeqabaqabeGadaaakeaadaaeWbqaaiabdofatjabdofatnaaBaaaleaadaWadaqaaiabdIha4bGaay5waiaaw2faaaqabaaabaGaemyAaKMaeyypa0JaeGymaedabaGaemOBa4ganiabggHiLdGccqGH9aqpcqaIXaqmcqaIZaWmcqGGUaGlcqaI5aqocqaI3aWncqaIWaamcqaIXaqmaaa@4142@

For the Joint Regression SS, both the numerator and denominator have been evaluated, which gives a value of 408.1129.

Values for both items in the Heterogeneity of Regression coefficients have now been calculated. Thus:

Heterogeneity of Regression coefficients = 408.5484 – 408.1129 = 0.4355.

To test if b_1 _and b_2 _are significantly different from one another, the Heterogeneity of regression coefficients SS is divided by ∑i=1n
 MathType@MTEF@5@5@+=feaafiart1ev1aaatCvAUfKttLearuWrP9MDH5MBPbIqV92AaeXatLxBI9gBaebbnrfifHhDYfgasaacH8akY=wiFfYdH8Gipec8Eeeu0xXdbba9frFj0=OqFfea0dXdd9vqai=hGuQ8kuc9pgc9s8qqaq=dirpe0xb9q8qiLsFr0=vr0=vr0dc8meaabaqaciGacaGaaeqabaqabeGadaaakeaadaaeWbqaaaWcbaGaemyAaKMaeyypa0JaeGymaedabaGaemOBa4ganiabggHiLdaaaa@33A6@ Residual SS

Where:

∑i=1n
 MathType@MTEF@5@5@+=feaafiart1ev1aaatCvAUfKttLearuWrP9MDH5MBPbIqV92AaeXatLxBI9gBaebbnrfifHhDYfgasaacH8akY=wiFfYdH8Gipec8Eeeu0xXdbba9frFj0=OqFfea0dXdd9vqai=hGuQ8kuc9pgc9s8qqaq=dirpe0xb9q8qiLsFr0=vr0=vr0dc8meaabaqaciGacaGaaeqabaqabeGadaaakeaadaaeWbqaaaWcbaGaemyAaKMaeyypa0JaeGymaedabaGaemOBa4ganiabggHiLdaaaa@33A6@ Residual SS = Residual SS[_b1_] + Residual SS[_b2_]

The ∑i=1n
 MathType@MTEF@5@5@+=feaafiart1ev1aaatCvAUfKttLearuWrP9MDH5MBPbIqV92AaeXatLxBI9gBaebbnrfifHhDYfgasaacH8akY=wiFfYdH8Gipec8Eeeu0xXdbba9frFj0=OqFfea0dXdd9vqai=hGuQ8kuc9pgc9s8qqaq=dirpe0xb9q8qiLsFr0=vr0=vr0dc8meaabaqaciGacaGaaeqabaqabeGadaaakeaadaaeWbqaaaWcbaGaemyAaKMaeyypa0JaeGymaedabaGaemOBa4ganiabggHiLdaaaa@33A6@ Residual SS is equal to the sum of the residual sum of squares from data sets 1 and 2. These are listed in the analysis of variance tables associated with the original two regression analyses (Table 3).

∑i=1n
 MathType@MTEF@5@5@+=feaafiart1ev1aaatCvAUfKttLearuWrP9MDH5MBPbIqV92AaeXatLxBI9gBaebbnrfifHhDYfgasaacH8akY=wiFfYdH8Gipec8Eeeu0xXdbba9frFj0=OqFfea0dXdd9vqai=hGuQ8kuc9pgc9s8qqaq=dirpe0xb9q8qiLsFr0=vr0=vr0dc8meaabaqaciGacaGaaeqabaqabeGadaaakeaadaaeWbqaaaWcbaGaemyAaKMaeyypa0JaeGymaedabaGaemOBa4ganiabggHiLdaaaa@33A6@ Residual SS = 3.03 +2.30 = 5.33

The degrees of freedom for each individual residual SS are (n-2) which equals 5, where n is the number of points plotted on the calibration curve. Thus, where n is the same between the two regressions, as in this worked example, the degrees of freedom relating to the ∑i=1n
 MathType@MTEF@5@5@+=feaafiart1ev1aaatCvAUfKttLearuWrP9MDH5MBPbIqV92AaeXatLxBI9gBaebbnrfifHhDYfgasaacH8akY=wiFfYdH8Gipec8Eeeu0xXdbba9frFj0=OqFfea0dXdd9vqai=hGuQ8kuc9pgc9s8qqaq=dirpe0xb9q8qiLsFr0=vr0=vr0dc8meaabaqaciGacaGaaeqabaqabeGadaaakeaadaaeWbqaaaWcbaGaemyAaKMaeyypa0JaeGymaedabaGaemOBa4ganiabggHiLdaaaa@33A6@ Residual SS is calculated as (n-2)*2, which equals 10. This Residual SS characterises that proportion of the variation in y that is not dependent upon x for both regression models. Thus, the Residual SS is a measure of the amount of variation left over in the experiment, which is not accounted for by both models.

An F-variance ratio test can then be used to test if the variance associated with the Heterogeneity of regression coefficients SS is significantly greater than the variance associated with the Residual SS, as shown in Table 4.

For this test, the null hypothesis (H_0_) assumes that there is no significant difference between the two regression coefficients. The P value then represents the probability that the difference between the two regression estimates is purely due to chance. Adopting common statistical probability threshold values, if the P value is below 5% (P < 0.05) the null hypothesis is rejected. If P < 0.05 the alternative hypothesis is accepted, and the difference between the two regression estimates is real and the estimates are significantly different. In the example illustrated above, the P value was equal to 0.39, which indicated that the regression coefficients associated with the calibration curves produced by the two RT-QPCR platforms were not significantly different from one another.

This objective comparison between two calibration curves is useful as the statistical test takes into account the majority of those variables inherent in normal regression analysis. For example, the test uses the regression sum of squares associated with both calibration curves, which represents that part of the variation in the dependent variable (Ct) that is accountable for by the independent variable (log of the DNA concentration). Thus, the regression sum of squares is a measure of how well the linear regression model fits the experimental data. In addition, the test takes into account the residual sum of squares associated with the calibration curves, which is an estimate of the amount of variation remaining in the experimental data that is not explained by the linear regression model. Because the number of degrees of freedom associated with such items as the heterogeneity of regression coefficients sum of squares and the joint remainder sum of squares can be calculated, variance estimates can be made. These estimates then facilitate an F variance ratio test that can be used to predict the probability that differences arise between the two regression coefficients due to chance alone.

Based on conventional RT-QPCR theory [42], the regression coefficient and the PCR amplification efficiency are related according to the following equation:

b=−(1logE)
 MathType@MTEF@5@5@+=feaafiart1ev1aaatCvAUfKttLearuWrP9MDH5MBPbIqV92AaeXatLxBI9gBaebbnrfifHhDYfgasaacH8akY=wiFfYdH8Gipec8Eeeu0xXdbba9frFj0=OqFfea0dXdd9vqai=hGuQ8kuc9pgc9s8qqaq=dirpe0xb9q8qiLsFr0=vr0=vr0dc8meaabaqaciGacaGaaeqabaqabeGadaaakeaacqqGIbGycqGH9aqpcqGHsisldaqadaqaamaalaaabaGaeeymaedabaGaeeiBaWMaee4Ba8Maee4zaCMaeeyraueaaaGaayjkaiaawMcaaaaa@3798@

where E = efficiency of PCR reaction. Thus any differences observed between the regression coefficients associated with the two calibration curves will also be related to the two PCR amplification efficiencies associated with the data sets. Small changes in both E, and hence b can influence results substantially, hence it is important that b and E are compared in some way. Whilst this aspect has been recognised for some time, no advice has been given regarding a statistical or objective test on how to achieve this for RT-QPCR studies. The test described in this paper shows one potential way of meeting this requirement.

The above technique may be usefully applied in further situations to objectively compare the gradients associated with two calibration curves. For example, it would be applicable to use the statistical approach in relation to historical data sets. A calibration curve based on a current data set may be compared to a calibration curve conducted six months previously. This may be conducted in order to qualify if a reference material is behaving comparably between the two time points. Any significant differences between the two calibration curves may be due to stability or other issues, and are worth investigating further before using the reference material for any additional analytical work. Furthermore, the technique for comparing regression coefficients can be implemented when examining two calibration curves that have been produced using methods that are similar except for one factor. For example, the statistical technique can be applied when standard curves are produced from the same analytical standards but use different real time PCR platforms. In this example, the scientist may wish to compare the calibration curves between the platforms in order to determine if they are essentially giving the same estimates. Additionally, the technique can be applied when the sources of the standards used to produce the calibration curve are different. For example, calibration curves on the same real-time PCR platform may be produced using plasmid or genomic DNA which have been quantified and diluted to the same concentration. The analyst may then want to objectively compare the gradient associated with each of the calibration curves in order to determine statistically if they are operating to the same efficiency. More mundane, but no less important applications of the technique would involve examining the relationship between two calibration curves based on replicate runs of the same experiment in order to determine if significant differences occur.

### Limitations of approach and comparability with other methods

The test described above has been explained in detail, to facilitate its implementation using only the basic functions found in standard spreadsheet software. Similar results can also be achieved if more specialised statistical software is available to the analyst. However, knowledge of statistical expressions, and familiarity regarding terminology that may be specific to the software package is often assumed.

Statistical tests for the difference between regression coefficients can be implemented using an analysis of covariance approach through a general linear model. [43,44]. The specific details concerning the theory behind this model can be found in [45], but a brief description of the application of the analysis of covariance is given here. The general linear model can be used to test three hypotheses: that the two gradients are significantly different from zero; that the gradients of the two lines are the same (heterogeneity of regression coefficients); and the hypothesis of equality of intercepts (the point where both lines cross the y-axis is the same). Using statistical software this can be achieved by specifying the Ct value as the response variable and the log (copy number) as the covariate. In addition, the model used to make the comparison is specified as the interaction between the two variables. Using data sets 1 and 2, an analysis of covariance results table is shown in Table 5.

The null hypothesis associated with the 'Intercept' item is that there are no significant differences between the intercepts associated with the two regression lines. As the P value is not significant, it is accepted that any differences observed between the intercepts associated with both lines was purely due to chance alone.

With reference to the 'Slope' item, the null hypotheses states that the gradients are not significantly different from zero. As the associated P value indicates the test is statistically significant, it is accepted that the gradients associated with the two regression lines was different from zero.

Finally, the null hypothesis associated with the 'Heterogeneity of regression coefficients' item is the same as the original test described above. This specifies that there are no significant differences between the two estimates of the regression coefficients. The P value associated with this item is non-significant, therefore the null hypothesis is accepted that there are no differences between the two gradients. The degrees of freedom, sum of squares, mean square estimates, F and P values associated with the basic test described above, and the analysis of covariance using statistical software are exactly the same.

The test for comparing regression coefficients, as described in this paper, is applicable for two calibration curves. The technique can be further extended to three or more calibration curves by computing additional statistics inclusive of the sum of squares and sum of products for the additional data sets, and taking into account the joint remainder sum of squares associated with all data sets. Additionally, the technique is potentially applicable to data sets which have been measured at different intervals and points for the independent x variable. However, care must be taken in this respect, as the interpretation of the results may be further complicated as comparisons are not independent of each other. Such non-orthogonal comparisons can render statistical conclusions invalid. Due to the complexity involved in these further calculations, the use of specific statistical software in order to conduct the analysis of covariance using the general linear model is suggested. Additionally, because of the multiplicity of testing between pairs of regression lines, the chance of error increases so a more stringent significance level should be adopted. These further extensions to the technique are discussed in [39-41,43-45].

## Conclusion

RT-QPCR has become established as a benchmark in many areas for trace detection of DNA, and is increasingly being used in legislative and regulatory enforcement. Because of the increased focus on the technique, it is of paramount importance that all factors that can contribute to significant measurement uncertainty in the end result of the assay are identified. Currently, most of this evaluation of measurement uncertainty has concentrated on the experimental work associated with the RT-QPCR process, and little work has been done to investigate the uncertainty at the data handling and processing stage. Previous work [2] identified several aspects associated with the data handling stage that can cause variation in the end result unless standard guidelines are adopted for their implementation. The current paper highlights two additional aspects of data handling that can potentially contribute towards significant measurement uncertainty in the final outcome.

The first aspect involved the identification of potential outlying values associated with a data set, followed by their correct characterisation via an objective statistical test and recommended acceptance or rejection criteria according to ISO guidelines. The second aspect involved an objective test to calculate the probability that two calibration curves were statistically different, enabling the use of a more quantitative approach to comparing regression coefficients than have been previously reported.

The approaches described in this paper illustrate preliminary methods with which to standardise the reporting of results. As is inherent with the application of any statistical tool, these approaches have limitations but they serve to emphasise additional areas where more work needs to be conducted in order to standardise the data handling aspects associated with RT-QPCR assays. Additionally, the approaches are not unique, but their application to the area of real-time PCR is novel.

The focus of the current study is the implementation of statistical techniques in order to examine some factors that can account for variability in results associated with RT-QPCR measurements. This statistical analysis of data helps contribute towards a greater understanding of some of the areas of uncertainty involved in RT-QPCR.

Additional studies have detailed the mathematics associated with RT-QPCR, involving discussions on the use of cycle threshold values and the precision associated with replicate standard curves [21]. Whilst linear standard curves are often used as calibration curves, a recent study has suggested an approach utilising a sigmoidal function to model fluorescence data as an alternative, in order to increase the reliability of RT-QPCR measurements [22]. A further study provides an in-depth review regarding some of the factors that can account for significant variability in RT-QPCR measurements including template, operator, data analysis and subsequent reporting of results [25]. Thus, in any approach to aid in standardisation of RT-QPCR measurements, it is the summation of many studies inclusive of statistics, mathematical modelling, technical and practical approaches that cumulatively provide a better understanding of all components that can add significant uncertainty to a result.

To fully realise and explore the harmonisation of data handling regarding RT-QPCR assays requires collaborative efforts between scientists that routinely conduct the assays and can define the problem, and statisticians that can suggest the optimal approach. It is hoped that by describing these two aspects in detail in this paper, it can be seen that further variation at the data handling stage can contribute to uncertainty in the end result. The procedures outlined in this paper thus have the potential to contribute towards providing a set of standardised guidelines for the handling and processing of data arising from RT-QPCR methods, to enable a more systematic and controlled approach to interpretation of the end result.

## Methods

### Experimental work

The data was generated by singleplex RT-QPCR 5'-3' exonuclease assay using two real-time platforms: the DNA Engine Opticon 2^® ^(MJ Research, Inc., USA) and MX3000P Real-Time PCR System (Stratagene, USA). These platforms are primarily used to detect the exponential accumulation of fluorescent moieties arising from PCR-based amplification of a target sequence.

The RT-QPCR assay employed within this study targets the human glyceraldehyde-3-phosphate dehydrogenase (GAPDH) gene and was adapted from a previously developed assay system [46].

The study comprised three replicate analysis plates per platform. Each plate contained seven standards (31623, 10000, 3162, 1000, 316, 100 and 32 genomic equivalent copies) and two sample unknowns (562 and 5623 genomic equivalent copies) for evaluation. All standards and samples within a plate were replicated six times. The standards and sample unknowns were generated by serial dilution from the same source stock, and were devised to ensure equal spacing of the calibration data points when plotted on a logarithmic scale for linear regression analysis.

Amplification reactions (25 μl) were performed using 1 × Absolute™ QPCR dUTP Mix (ABgene, UK) supplemented with 100 nM ROX (passive reference dye), 450 nM GAPDH-FWD and GAPDH-REV primers, 225 nM GAPDH-Probe (FAM and TAMRA labelled probe), and variable concentrations (expressed in genomic equivalents) of Human Genomic DNA: Female (Promega, UK). Primers and probes were supplied by Sigma-Genosys (UK).

Reactions were run on the two platforms using the following thermal cycling parameters: 50°C for 2 min, 95°C for 10 min and 60 cycles of 95°C for 15 s, 60°C for 1 min. RT-QPCR reactions were performed in accordance with the equipment manufacturer's instructions (MX3000P™ Real-Time PCR System On-line Help; DNA Engine Opticon™ System Operations Manual) and reagent manufacturer's instructions (Absolute™ QPCR Mixes product insert- Abgene 2003).

Analyses were performed in accordance with the manufacturer's recommendations using platform specific software: MX3000P v1.01 (MX3000P Real-Time PCR System) and Opticon Monitor™ v1.03 (DNA Engine Opticon 2^®^). Statistical analysis of the data was performed using Statistica 6.0 software (Statsoft Inc., USA).

### Preliminary statistical analysis

For each of the two platforms independently, a two-way analysis of variance (ANOVA) was used to test for significant differences. The two factors examined were plates and samples (results not shown). The results from both platforms showed that at the 95% level of confidence, there was a non-significant interaction (P > 0.05) between the samples and the three replicate plates within a platform, implying that the samples were behaving consistently between the plates. This gave sufficient statistical justification in order to pool the three plates within a platform together so that each sample was represented by 18 replicates.

### Outlier testing

Data produced from both platforms was tested for outlying values. Data set 3 was derived from the experimental data set and consisted of three selected samples from the Opticon 2 platform: sample X (316 copies of genome), sample Y (562 copies of genome) and sample Z (5623 copies of genome). Each sample within Data set 3 was represented by a replication factor of 18. Data set 3 was used to illustrate the application of the outlier test.

### Comparing regression coefficients

Data sets 1 and 2 represented data from the seven standards used to produce calibration curves in the experimental data set from the MX3000P and Opticon 2 platforms respectively. These data sets were used for comparing regression coefficients based on the calibration curve produced by each platform. Each of the seven standards was replicated 18 times.

## Authors' contributions

GJN conceived of the study and conducted all experimental laboratory work to generate the data. MJB advised on the experimental work, carried out the statistical analysis and modeling associated with the data, and drafted the manuscript. CAF managed the project and coordinated all activities. NH provided additional background material and helped to draft the manuscript. All authors read and approved the final manuscript.

## Figures and Tables

**Table 1:** Statistical identification of outliers on data set 3. Data set 3 comprised of three samples X, Y, and Z. Each sample consisted of 18 replicates of known analyte concentration. The table lists the response values (Ct) associated with these 18 replicates per sample, as well as indicating which values appeared to be potential outliers according to the box and whisker plots. For each sample, the Grubbs' test statistic is computed, as well as the classification and recommendations according to ISO guidelines.

	**Sample**		
	**X**	**Y**	**Z**
	24.24	24.41	22.97
	23.97	27.21	22.93
	24.44	27.02	22.95
	24.79	26.81	23.12
	23.92	26.64	23.59
	24.53	27.63	23.37
	24.95	28.42	24.17
	24.76	25.16	23.48
	25.18	28.53	23.80
	25.14	28.06	23.43
	24.57	27.77	23.66
	24.49	28.74	28.79
	24.68	28.35	23.77
	24.45	28.80	23.98
	24.48	27.99	23.56
	24.30	28.21	22.80
	24.60	28.00	23.29
	24.57	28.21	23.86

**Potential outlier value**	23.92	24.41	28.79
**Grubbs' test statistic**	1.89	2.63	3.83
**Outlying value classification**	Non significant	Straggler	Outlier
**Recommendations**	Accept	Accept	Reject

**Table 2:** Description of data sets 1 and 2 used for comparing regression coefficients. Data sets 1 and 2 were produced based on measuring the instrument response (Ct values) in relation to a range of standards of known copy number. The copy numbers of these standards are displayed in the first column, with the log to the base 10 of these copy numbers displayed in the second column. The Y_1 _column shows the Ct values corresponding to data set 1 from the first RT-QPCR platform, whilst the Y_2 _columns shows the Ct values corresponding to data set 2 from the second RT-QPCR platform.

**DNA copy nu**	**Log (DNA copy nu)**	**Y**_**1**_	**Y**_**2**_
31628	4.5	18.79	20.93
10000	4	20.47	22.74
3162	3.5	22.31	24.56
1000	3	25.44	27.31
316	2.5	28.74	30.11
100	2	32.24	33.38
32	1.5	34.82	36.37

**Table 3:** ANOVA table associated with simple linear regression analysis of data sets 1 and 2.

**Data set 1**					
**Item**	**df**	**SS**	**MS**	**F**	**P**
Regression	1	217.61	217.61	358.77	<0.001
Residual	5	3.03	0.61		
Total	6	220.64			
					
**Data set 2**					
**Item**	**df**	**SS**	**MS**	**F**	**P**

Regression	1	190.94	190.94	415.67	<0.001
Residual	5	2.30	0.46		
Total	6	193.24			

**df**: degrees of freedom; **SS**: sum of squares; **MS**: mean squares: **F**: F variance ratio; **P**: probability associated with F variance ratio.

**Table 4:** ANOVA table associated with heterogeneity of regression coefficients using basic spreadsheet software.

**Item**	**df**	**SS**	**MS**	**F**	**P**
Heterogeneity of regression coefficients	1	0.436	0.436	0.817	0.387263
Residual	10	5.33	0.533		

**df**: degrees of freedom; **SS**: sum of squares; **MS**: mean squares: **F**: F variance ratio; **P**: probability associated with F variance ratio.

**Table 5:** Table associated with analysis of covariance using advanced statistical software.

**Item**	**df**	**SS**	**MS**	**F**	**P**
Intercept	1	0.190	0.190	0.357	0.563273
Slope	1	408.113	408.113	765.766	<0.000001
Heterogeneity of regression coefficients	1	0.436	0.436	0.817	0.387263
Error	10	5.329	0.533		

**df**: degrees of freedom; **SS**: sum of squares; **MS**: mean squares: **F**: F variance ratio; **P**: probability associated with F variance ratio.
